# From Primary Care to Super Specialty: A Comparison of Workforce Expansion and Capacity Building in Internal Medicine in India and the United States

**DOI:** 10.7759/cureus.77221

**Published:** 2025-01-10

**Authors:** Nirupam Nadella, Lokesh Edara

**Affiliations:** 1 Department of General Medicine, Dr. D. Y. Patil Medical College, Hospital & Research Centre, Dr. D. Y. Patil Vidyapeeth (Deemed to be University), Pune, IND; 2 Department of Internal Medicine, Western Michigan University, Kalamazoo, USA

**Keywords:** capacity building, chronic illness care, doctor-patient ratio, healthcare disparities, internal medicine, medical education, postgraduate training, residency programs, superspecialty training, workforce expansion

## Abstract

Internal medicine (IM), a cornerstone of healthcare, focuses on diagnosing, treating, and preventing adult diseases. This review explores the training pathways and opportunities in IM in India and the United States (USA), highlighting critical disparities in postgraduate medical education. A combination of statistical analysis, literature review, and policy evaluation was utilized. Data were compiled from government databases, academic literature, and organizational reports to identify the number of postgraduate seats and training opportunities. Comparative metrics, such as per capita seat availability and training ratios, were analyzed to highlight disparities. In India, IM residencies, offered as Doctor of Medicine (MD) or Diplomate of National Board (DNB) programs, account for only a small proportion of postgraduate seats, with significant variations in per capita availability compared to the USA. While the number of undergraduate medical seats in India has grown substantially, postgraduate training opportunities, especially in IM and its subspecialties, remain limited. This discrepancy contributes to a shortage of internists and specialists, particularly in rural and underserved regions, impacting healthcare delivery. The USA offers a more robust IM training framework, with higher per capita residency and fellowship positions, ensuring a more comprehensive distribution of specialists. In contrast, India's lower per capita availability of IM seats has led to an imbalance in the doctor-to-patient ratio and prompted many Indian medical graduates to seek training abroad, resulting in a "brain drain." Addressing this disparity requires a systemic approach, including increasing postgraduate and superspecialty seats, adopting innovative training models, and aligning India's medical education system with global standards. Expanding training capacity, particularly in underserved regions, and transitioning to a residency-based model for certain specialties could improve healthcare outcomes and bridge the gap in IM expertise between India and developed nations.

## Introduction and background

Internal medicine is a medical specialty focused on the prevention, diagnosis, and management of diseases affecting adults through non-surgical methods. Physicians in this field often serve as primary care providers, overseeing comprehensive patient care and coordinating treatments provided by other specialists when necessary [1]. Physicians trained in IM, often referred to as internists, play a pivotal role in comprehensive patient care. This review aims to explore the structure, training pathways, and opportunities available in IM residencies, subspecialties, and fellowships in both India and the United States (USA).

In India, IM residency programs are typically structured as a three-year postgraduate course following the completion of a Bachelor of Medicine and Bachelor of Surgery (MBBS). Residency programs may lead to the degrees of MD (Doctor of Medicine) and DNB (Diplomate of National Board). MD is the most common pathway, offered by medical colleges affiliated with universities and governed by the National Medical Commission (NMC) [2]. DNB is a parallel route regulated by the National Board of Examinations in Medical Sciences (NBEMS), considered equivalent to the MD but with a greater emphasis on hands-on training in accredited private and public hospitals [3].

In the USA, IM residency programs span three years and are regulated by the Accreditation Council for Graduate Medical Education (ACGME). Admission typically follows the completion of an MD or Doctor of Osteopathic Medicine (DO) degree [4].

## Review

Indian scenario

India has witnessed a 116.8% increase in MBBS seats, rising from 54,348 in 2014 to 1,17,825 in 2024, marking substantial growth in medical education capacity [3,5]. During the same period, 399 new medical colleges were established and now the country has 778, a phenomenal increase of 105.28% [6]. In 2014-15, there were over 24,416 postgraduate (PG) medical seats in government and private medical colleges nationwide [5]. The Board of Governors of the Medical Council of India (now replaced with the National Medical Commission (NMC)) gave its approval to increase post-graduate medical seats (broad specialty) in the 2020-21 session by 4,807 [7]. In the 2024-25 academic session, approximately 61,879 post-graduate medical seats (47,207 MD/Master of Surgery (MS) seats and 14,672 DNB seats) will be available for 67,218 MBBS pass-outs [3,8]. These are steps in the right direction.

The World Health Organization (WHO) has promulgated a desirable doctor-population ratio of 1:1000 [9]. India is approaching the WHO's recommended doctor-to-population ratio. The doctor-to-patient ratio is 1:1,075, which includes all categories of registered medical professionals such as undergraduate and postgraduate doctors, specialists, and clinical and non-clinical physicians, as recognized by the NMC. If we consider all registered traditional medicine practitioners and assume 80% availability of allopathic doctors, the doctor-to-patient ratio is approximately 1:834 [10]. This suggests significant progress towards meeting global healthcare standards, while also highlighting the role of various medical systems in the country’s healthcare framework.

Comparison of postgraduate seats in India and the USA

In 2024, the United States, with a population of 334.91 million, had a total of 219,639 active IM physicians. This equates to an overall density of 65.58 IM physicians per 100,000 people [11]. India, with a population exceeding 1.45 billion, is the most populous country in the world [12], and it is four times more populous than the United States. According to the NMC's Indian Medical Register, there are 1,349,570 doctors registered with various state medical councils [13]. However, the database is still being updated; also, specific data on the number of practicing IM physicians is not publicly available. In contrast, the USA has one IM physician for every 1,524.85 individuals [11].

Currently, India's overall allopathic doctor-to-patient ratio stands at 1:1,074.56, which is significantly lower than the doctor-to-patient ratio in the USA [13]. This disparity suggests that the ratio of IM physicians in India is far from comparable to that of the USA. To improve the standards of general medical care in India and align with global norms, it is essential to work towards achieving similar ratios, with a particular emphasis on expanding the workforce of IM physicians to address the needs of the growing population.

There is a significant shortfall in postgraduate medical seats in India, with only 61,879 available, revealing a pronounced imbalance across specialties. IM has 6168 seats (5059 MD and 1109 DNB), accounting for 5.23% of total undergraduate seats and 9.96% of total postgraduate seats as mentioned in Table 1 [6,8]. In comparison, the USA offers 10,261 IM residency positions, which accounts for 34.20% of the total undergraduate medical seats available in the country, and 26.65% of the 38,494 total residency positions across all specialties [14,15].

**Table 1:** Comparison of undergraduate medical seats, postgraduate residency capacities, superspecialty training, and internal medicine education in India and the United States

Category	India	United States
Undergraduate Medical Seats (MBBS)	117,825	30,000
Total Postgraduate Seats (Residency)	61,879	38,494
General/Internal Medicine Residency Seats	6,168	10,261
Superspecialty Seats (Post-PG)/Fellowship	3,188	6,491
General/Internal Medicine Seats (Without Superspecialty/Fellowship)	2,980	3,770

India currently has 3188 superspecialty seats in IM (1368 DM and 1820 DNB), leading to approximately 3308 doctors practicing IM post residency [8]. In the USA, there are 6491 fellowship spots for physicians who have completed an IM residency [14]. This results in an estimated 3770 physicians continuing to practice general IM [14,15].

India has a medical graduate per capita rate of 81.84 per million while the USA has a medical graduate per capita rate of 89.55 per million [8,15]. Even though the Indian per capita rate of medical graduates is close to the USA, some states like Bihar (22.9 per million), Jharkhand (40.12 per million), and Nagaland (50.57 per million) are lagging behind compared to the rest of India [8]. India with a population of 1440 million has PG seats (Residencies in the USA) equal to 61,789, which is equivalent to a per capita rate of 42.97 per million [4,8]. On the other hand, the USA with a population of 335 million has 38,494 PG seats (Residencies), which is equivalent to a per capita rate of 114.89 per million [16]. This is almost three-fold more than that of India. In the graduate seats per capita rate, India was nearly at par with the USA, but in the PG seats per capita rate, India falls behind. The Ministry of Health in India announced that it is working to equal the number of undergraduate and postgraduate medical seats within four years so that all MBBS graduates can pursue PG courses [17].

IM physicians, also called internists or doctors of IM, specialize in diagnosing complex medical conditions and managing chronic illnesses in adults [18]. Their expertise allows them to provide comprehensive, long-term care, often solving diagnostic challenges and handling multiple coexisting conditions. Known as "the doctor’s doctor," they are frequently consulted by other healthcare professionals for their ability to provide insights into complicated cases, making them crucial for both patient care and medical practice. Thus, IM plays a vital role in healthcare.

With a population of 1440 million as of April 2024 [12], India has 6,168 PG seats (equivalent to residency in the USA) in IM, translating to a per capita rate of 4.28 per million [3,8]. Similarly, the USA, which has a population of 335 million as of January 2024 [19], has 10,261 PG (or residency) seats in IM, translating to a per capita rate of 30.63 per million [14].

The inability to train enough professionals impedes India's ability to satisfy global health standards and effectively handle non-communicable illnesses, which are on the increase in the nation. The difference in PG training possibilities encourages Indian medical graduates to pursue residency programs in other countries like the USA [20]. While this improves their employment chances, it also contributes to India's trained healthcare professional shortage. The departure of doctors trained in India, sometimes known as the "brain drain," represents a huge loss of investment in medical education and training for the nation. India now has 3188 superspecialty seats in IM as mentioned in Table 2, at a per capita rate of 2.21 million [3,8]. In the USA, there are 6491 fellowship seats available for physicians who have completed an IM residency, which equates to 19.37 per million [16]. Table 2 provides a comparative overview of the training capacities in IM superspecialties and fellowships between India and the USA. It highlights the number of available seats in various subspecialties such as cardiology, gastroenterology, and endocrinology, showcasing the disparities in advanced medical training opportunities in both countries.

**Table 2:** Comparison of internal medicine superspecialty and fellowship training capacities: India vs. United States DM: Doctorate of Medicine; DrNB: Doctorate of National Board

Specialty	India (DM and DrNB seats)	Specialty	United States (Fellowship seats)
Pulmonary Medicine	42+0=42	Pulmonary Disease	31
Pulmonary Medicine & Critical Care	13+0 = 13	Pulmonary Disease & Critical Care Medicine	781
Neurology	356+237 = 593	Sleep Medicine	213
Medical Oncology	124+165 = 289	Oncology	3
Cardiology	154+352 = 506	Cardiovascular Disease	1199
Medical Genetics	4+4 = 8	Rheumatology	276
Critical Care Medicine	83+484 = 567	Critical Care Medicine	187
Medical Gastroenterology	231+220 = 451	Gastroenterology	690
Clinical Haematology	17+41 = 58	Haematology & Oncology	755
Clinical Immunology & Rheumatology	6+34 = 40	Allergy & Clinical Immunology	169
Infectious Disease	15+12 = 27	Infectious Disease	450
Nephrology	241+208 = 449	Nephrology	488
Paediatric Neurology	10+5 = 15	Hospice & Palliative Care	451
Endocrinology	46+58 = 104	Endocrinology, Diabetes & Metabolism	379
Hepatology	26+0 = 26	Geriatric Medicine	419

Rising healthcare demands, an aging population, and the increased complexity of medical problems all contribute to the growing need for more superspecialty seats in IM in India. As more people require specialized treatment for chronic and multiple disorders, there is a dearth of skilled specialists in sectors such as cardiology, gastrointestinal, and endocrinology. Expanding the number of superspecialty seats will assist in closing these gaps, resulting in higher-quality treatment and improved patient outcomes [21]. India is regarded as a prominent medical tourism destination because it can offer top-notch medical care at a far lower cost than other nations [22]. We require additional specialists and super specialists primarily to meet the healthcare needs of the Indian population. Once this gap is addressed, further expansion of specialist services could support medical tourism as well.

As shown in Table 3, in India, specialties like Respiratory Medicine, Geriatric Medicine, and Palliative Medicine are offered as MD (Residency) programs, leading to an increased number of specialists. Respiratory Medicine is unique in India, as it is available both as an MD (three years) and a DM (fellowship) course after IM residency [8]. This approach contrasts with the USA, where these specialties are fellowships following IM residency, ensuring a higher number of both specialists and internists in India [16]. The Indian model ensures broader access to these specialties. Conversely, disciplines like Sleep Medicine and Allergy & Clinical Immunology lack formal residency programs in India, with the latter being offered as a fellowship in the USA after IM or Pediatrics training [16]. In India, Neurology and Pediatric Neurology are three-year courses following an IM residency, totaling six years, whereas, in the USA, they are four-year residency programs [8,14].

**Table 3:** Comparison of direct residency specialties: United States fellowship specialties offered as direct residencies in India vs. Indian superspecialty courses recognized as direct residencies in the United States MD: Doctor of Medicine; DNB: Diplomate of National Board References: [3,8,16]

Specialty	India (MD + DNB )	Specialty	United States (Residency)
Pulmonology Medicine	5+0 = 5	Neurology	878
Geriartic Medicine	48+2=50	Child Neurology	184
Palliative Medicine	20+18=38		
Respiratory Medicine	186+108=294		

## Conclusions

The stark difference in the availability of PG residency seats in IM between India and the USA reveals significant systemic issues in healthcare workforce training and distribution, with far-reaching implications for both healthcare delivery and medical education. In India, the low per capita availability of postgraduate seats in IM suggests an insufficient capacity to train specialist physicians in comparison to the population's demands. As a result, there aren't enough qualified internists to deal with the rising burden of chronic and complicated illnesses, especially in rural and disadvantaged areas. In contrast, the USA's higher per capita availability suggests a stronger training system, allowing the country to retain well-distributed and appropriately educated IM practitioners. The scarcity of PG training seats in India brings an imbalance in doctor-patient ratios for specialized treatment. This can result in overcrowded healthcare systems, lengthier patient wait times, and degraded quality of service.

To increase the number of superspecialists and internists, we advocate that India transition to the MD (Residency) model for Neurology, Pediatric Neurology, and Endocrinology, rather than the present DM (fellowship) structure. This would raise the per capita rates for IM and superspecialty physicians. This adjustment might help handle the increased demand for experts while also assisting the Indian Health Ministry in balancing the number of postgraduate seats with undergraduate seats, therefore improving healthcare results.

## References

[1] (2024). National Cancer Institute: Internal medicine. https://www.cancer.gov/publications/dictionaries/cancer-terms/def/internal-medicine.

[2] (2024). National Medical Council: PG medical examination regulations, 2000. https://www.nmc.org.in/rules-regulations/p-g-medical-education-regulations-2000/#:~:text=Postgraduate%20Medical%20Education%20in%20broad,with%20the%20exceptions%20wherever%20indicated.

[3] (2024). National Board of Examinations: Department of accredition. https://accr.natboard.edu.in/online_user/frontpage.php.

[4] (2023). Accreditation Council for Graduate Medical Education. Official website of the Accreditation Council for Graduate Medical Education. ACGME Program Requirements for Graduate Medical Education in Internal Medicine.

[5] (2014). Press Information Bureau (PIB). Government of India approves the establishment of 11 new medical colleges. MBBS and PG Seats in Medical Institutions.

[6] (2024). National Medical Commission: List of colleges teaching MBBS. https://www.nmc.org.in/information-desk/for-students-to-study-in-india/list-of-college-teaching-mbbs/.

[7] (2024). Hindustan Times: MCI approves increase in PG medical seats by 4800 in 2020-21. Minutes of the Meeting of the Board of Governors - Appointed Expert Group Meeting.

[8] (2024). National Medical Commission: College and course search. https://www.nmc.org.in/information-desk/college-and-course-search/.

[9] Kumar R, Pal R (2018). India achieves WHO recommended doctor population ratio: a call for paradigm shift in public health discourse!. J Family Med Prim Care.

[10] Mandaviya Mandaviya, M M (2024). The Economic Times: Doctor population ratio in country stands at 1: 834, Mansukh Mandaviya tells Lok Sabha. https://economictimes.indiatimes.com/news/india/doctor-population-ratio-in-country-stands-at-1834-mansukh-mandaviya-tells-lok-sabha/articleshow/107561323.cms?from=m.

[11] (2024). American Board of Internal Medicine (ABIM). Candidates Certified: All Candidates. Philadelphia: American Board of Internal Medicine. Candidates Certified - All Candidates.

[12] (2024). Business Standard: India's population estimated at 1.4 bn, 24% in 0-14 age bracket: UNFPA. https://www.business-standard.com/economy/news/india-s-population-estimated-at-1-4-bn-24-in-0-14-age-bracket-unfpa-124041700171_1.html.

[13] National Medical Commission (NMC). Indian Medical Register. New Delhi: National Medical Commission (2024). National Medical Commission (NMC). Indian Medical Register. New Delhi: National Medical Commission. MBBS Doctor-Population Ration [Lok Sabha Starred Question No. 80].

[14] (2024). National Resident Matching Program Results and data : 2024 main residency match. National Resident Matching Program Results and Data: 2024 Main Residency Match®.

[15] (2024). International Medical Aid: How many medical schools in US: the definitive guide (2024). https://medicalaid.org/how-many-medical-schools-in-us-the-definitive-guide/.

[16] (2022). National Resident Matching Program. 2022 Specialties Matching Service: Match Results and Data. Washington, DC: National Resident Matching Program. National Resident Matching Program, Results and Data: Specialties Matching Service 2022 Appointment Year.

[17] Mandaviya M (2024). Shiksha: Govt trying to even UG, PG medical seats in 4 years: Mansukh Mandaviya. Shiksha.

[18] Loscalzo J, Fauci A, Kasper D, Hauser S, Longo D, Jameson JL (2022). Harrison's Principles of Internal Medicine, 21e. https://accessmedicine.mhmedical.com/content.aspx?bookid=3095&sectionid=259856983.

[19] (2024). US Department of Commerce: Census Bureau projects U.S. and world populations on new year’s day. https://www.commerce.gov/news/blog/2024/01/census-bureau-projects-us-and-world-populations-new-years-day#:~:text=As%20the%20nation%20rang%20in,Day%20(April%201)%202020.

[20] Kansal R, Singla A, Bawa A (2023). A nationwide survey on the preference of Indian undergraduate medical students to go abroad for higher studies and residency. J Family Med Prim Care.

[21] Supe A, Burdick WP (2006). Challenges and issues in medical education in India. Acad Med.

[22] Hazarika I (2010). Medical tourism: its potential impact on the health workforce and health systems in India. Health Policy Plan.

